# Distinct forms of motion sensitivity impairments in Alzheimer’s disease

**DOI:** 10.1038/s41598-019-48942-3

**Published:** 2019-09-10

**Authors:** Yining Liu, Depeng Feng, Hui Wang

**Affiliations:** Department of Neurology, Liaocheng People’s Hospital, Shandong Province, 252000 China

**Keywords:** Human behaviour, Neurological disorders

## Abstract

Motion sensitivity impairment in Alzheimer’s disease (AD) is often characterized as elevated coherence threshold. An alternative way to measure motion sensitivity is the direction threshold, i.e., the minimal angle of motion direction that can be discriminated. So far, it is less clear whether and how the direction threshold is altered in AD. Here we asked a group of AD patients and two control groups of healthy participants (young and elderly adults) to judge their perceived heading direction based on a field of optic flow stimuli simulating a forward translation in the environment. We manipulated the heading direction and the coherence of the optic flow independently and measured the direction and coherence thresholds from each participant. We found that the direction threshold increased significantly in AD patients as compared to healthy controls, like the coherence threshold. Yet, the elevation in the direction threshold was less pronounced than the coherence threshold. Moreover, the magnitudes of the direction and coherence thresholds in AD patients were not correlated. Our results suggest that coherence and direction impairments are two distinct forms of motion deficits in AD patients which might be associated with independent neural mechanisms.

## Introduction

Alzheimer’s disease (AD) is a prevalent neurodegenerative disease affecting the quality of life of millions of elderly adults^1^. Besides the conventional symptom of cognitive declines, AD patients often show visual perceptual deficits that are generally thought to be associated with dysfunctions in the dorsal visual stream of the brain^2–4^. Many behavioral studies have demonstrated that AD patients exhibited impaired motion processing^5–7^ and visuospatial disorientation^8–10^, implicating the involvement of posterior parietal regions in the dorsal visual system. The dorsal stream hypothesis of AD was also supported by converging evidence showing parietal atrophy^11–13^ and reduced activities in the superior parietal lobule^14–16^ in the AD group as comparing to the healthy control group.

Previous studies have attempted to identify visual motion perceptual deficits in AD. In these studies, the investigators focused mainly on comparing the motion coherence threshold between AD patients and healthy control participants^5–8,17,18^. Specifically, a proportion of randomly moving dots was added to a cloud of coherently moving dots and subjects were asked to report the perceived motion direction (e.g., leftwards vs. rightwards) while the coherence of the motion stimulus was manipulated^19,20^. It was found that AD patients in general had larger motion coherence thresholds than healthy elderly individuals, and this held true for the perception of simple horizontal motion^5–7^ and particularly complex optic flow pattern^8,17,18^ simulating self-motion in the environment (heading). Since the elevation of motion coherence threshold occurs for both simple motion and optic flow, it is unclear whether AD pathology reflects a global processing deficit in general, or a specific heading deficit that could be regarded as visuospatial disorientation.

In the study of optic flow perception, the typical way to measure heading sensitivity was to assess the heading direction threshold, i.e., the smallest heading angle that could be discriminated against a reference heading direction (e.g., the straight-ahead direction)^21–23^. In these studies, the heading direction was varied from trial to trial but usually there was no visual noise added in the optic flow stimuli (100% coherence). The coherence threshold and direction thresholds are two common measures of optic flow sensitivity, but they may emphasize on the different aspects of visual processing^24^. The coherence threshold is generally considered to probe the global processing ability of the visual system, because it requires integrating signal and noisy moving dots to form a unified motion perception^25,26^. In contrast, the direction threshold estimated in a noise-free stimulus might focus more on the fine direction discrimination ability of the perceptual system. So far, most studies in AD research explore the coherence threshold^5–8,17,18^; it is much less understood whether and how the direction threshold might be altered^6^. To date, there is a lack of attempt in the literature to investigate the possible link between the coherence impairment and heading directionality impairment in AD patients.

The main goal of this study is to explore the characteristics of the direction threshold in AD patients and its relation to the impaired coherence threshold. Specifically, we will present AD and healthy control participants with optic flow stimuli and measure their direction and coherence thresholds. In doing so we can achieve the following three goals: first, we can validate our study by replicating the previous findings on the coherence threshold impairments in AD; second, we can explore whether and how the direction threshold is altered in AD; third, we can investigate the possible link between the coherence and direction threshold impairments in AD patients. Altogether, this study will strengthen and extend our current understandings of the abnormalities of motion perception in AD, and will shed important new lights on the cortical basis of motion deficits associated with AD pathology.

## Materials and Methods

### Subjects

The current study involved forty-eight human participants in total. These participants were recruited by advertisement and were divided into three groups (each group with sixteen subjects). The AD group was recruited from clinically diagnosed AD patients in Liaocheng People’s Hospital. They met the National Institute of Neurological and Communicative Disorders and Stroke–Alzheimer’s Disease and Related Disorders Association criteria (NINCDS-ADRDA)^27^ for probable AD, and additionally scored less than twenty-six points in the Mini Mental State Exam (MMSE)^28^. Ten of these AD patients scored between twenty and twenty-five points in the MMSE test (mildly impaired) and the remaining six patients scored between twelve and twenty points (moderately impaired). The mean and standard deviation of the MMSE scores for all AD patients were 20.1 ± 4.8. The other two groups comprised healthy control individuals. The elderly control group consisted of sixteen healthy elderly whose age and sex matched the AD group. The mean and standard deviation of age were 63.9 ± 5.6 years for the elderly group and 65.7 ± 7.2 years for the AD group. Most of the elderly subjects were the spouses of AD patients. The young group was healthy young students from local universities (mean ± SD: 22.1 ± 5.5 years old). All subjects had normal or corrected-to-normal vision and were free of vision-related eye diseases according to pre-experiment screening. The young participants were screened based on self-reported questionnaire. The elderly and AD patients were examined with ophthalmological tests. All participants have provided an informed written consent for their participation in the study. For healthy participants (the two control groups), the written consent was obtained directly from each participant, and for AD patients, the written consent was obtained from their spouses or relatives. All the protocols for consent agreement and experimental procedures described in the following section were in full accordance with the local guidelines approved by the Ethics Review Committee of Liaocheng People’s Hospital, Shandong Province.

### Optic flow stimuli

**Figure 1:**
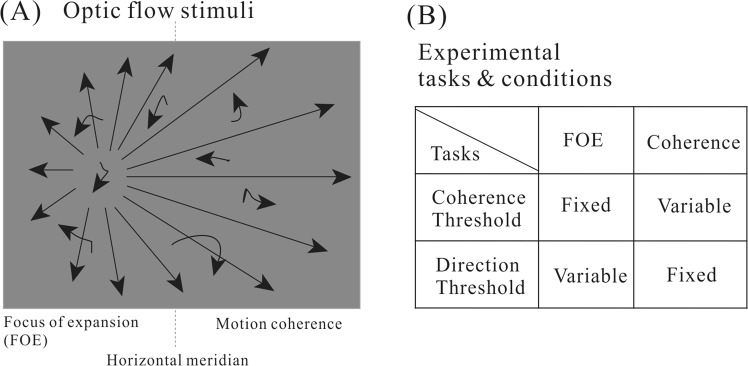
The task design. (**A**) Optic flow stimuli had a focus of expansion (FOE) corresponding to the direction of self-motion (leftwards in this case). Motion coherence (percentages of dots moving coherently) was also manipulated. (**B**) Two task conditions. In the coherence threshold task, motion coherence was varied while FOE was fixed; in the direction threshold task, FOE was varied while motion coherence was kept at 100%.

Subjects were instructed to sit comfortably in a dimly-light room. They rested their heads on a chinrest, which helps to minimize head movements. The chinrest was placed 57 cm away from a Dell monitor (resolution: 1024 × 768 pixels; refreshing rate: 100 Hz). Subjects viewed the monitor binocularly. We used the Psychophysics Toolbox^29,30^ to generate the optic flow stimuli. The stimuli consisted of a field of 3D moving dots mimicking the optic flow pattern experienced during self-motion (Fig. 1A). The size of the optic flow stimuli subtended 38.7 deg horizontally and 29.5 deg vertically. There were a total of 1600 white moving dots (32.7 cd/m^2^) on a dark background (4.6 cd/m^2^). Each dot had a diameter of 0.1°. The dots were moving in a virtual trapezoidal volume simulating a forward translation of 2.3 m/s in the environment. Dots that travelled outside the trapezoidal volume were replaced to new random positions on the starting base. There were two types of moving dots: the signal dots moving coherently in a specific direction and noisy dots moving in random directions. The percentage of coherently moving dots were referred to as the motion coherence. The optic flow had a focus of expansion (FOE) which corresponded to the heading direction of self-motion. The position of FOE was varied along the horizontal meridian on the screen. The motion coherence and heading direction (FOE) will be manipulated depending on the specific task condition.

### Experimental protocols

There were two task conditions (Fig. 1B). In the coherence threshold task, the FOE was kept at 8 degrees, and was either to the left or the right of the screen center. The motion coherence was varied from trial to trial to measure the coherence threshold. Motion coherence in each trial was randomly chosen from the following six values: 0%, 6.25%, 12.5%, 25%, 50%, 100%. In the direction threshold task, the motion coherence was fixed at 100% while the heading direction FOE was manipulated to estimate the direction threshold. The FOE was varied among ten possible values: ±0.5°, ±1°, ±2°, ±4°, ±8°. Positive values mean that the FOE was to the right side of the center of the screen and negative values on the left side. The order of the coherence threshold task and direction threshold task was balanced across participants in each group. Each task condition was repeated 10 trials, and the manipulated variable in each task (FOE or coherence) was randomized from trial to trial. We used the method of constant stimuli to obtain the psychometric performance. We decided the threshold as the point of 80% performance in each task condition. Each participant had to perform a total of 220 trials, which typically lasted about thirty minutes. They were allowed to take breaks that are either within a task or between tasks.

On each trial, participants were required to fix their gazes at the screen center (a cross) for 0.5 s. Then the optic flow stimuli were presented for 1 s while the eye fixation was maintained. After stimulus offset, they were required to report the perceived heading direction (left vs. right relative to the screen center) by pressing one of the two buttons on the keyboard. No feedback on the correctness of the trial was provided. Before experimentation, each participant was given the opportunity to practice for some trials with the feedback, allowing them to familiarize with the task.

### Data analyses and statistics

The data analysis and statistics were carried out using self-programed MATLAB scripts. For each participant in each task condition, we computed the percentage of correct response as a function of either direction or coherence to obtain the raw psychometric curve. We then fitted a cumulative Gaussian function to each psychometric curve. We decided the threshold as the point of 80% performance. These coherence and direction thresholds were then pooled across participants in each group for statistical analysis and comparisons. We conducted two levels of statistical testing on the thresholds. On the first level, we performed two separate, one-way independent ANOVA analysis, to test the group effects on the coherence and direction thresholds, respectively. On the second level, we combined the two thresholds by normalizing them to the mean threshold in the young adults. The combined analysis enabled us to conduct a two-way mixed model ANOVA of the thresholds (between-subject factor: elderly vs. AD; within-subject factor: normalized coherence and direction thresholds. In addition, we conducted Pearson’s linear correlation to test if the coherence and direction thresholds were correlated in each group. We set the level of significance at 0.05 for all statistical analysis in this study.

## Results

**Figure 2:**
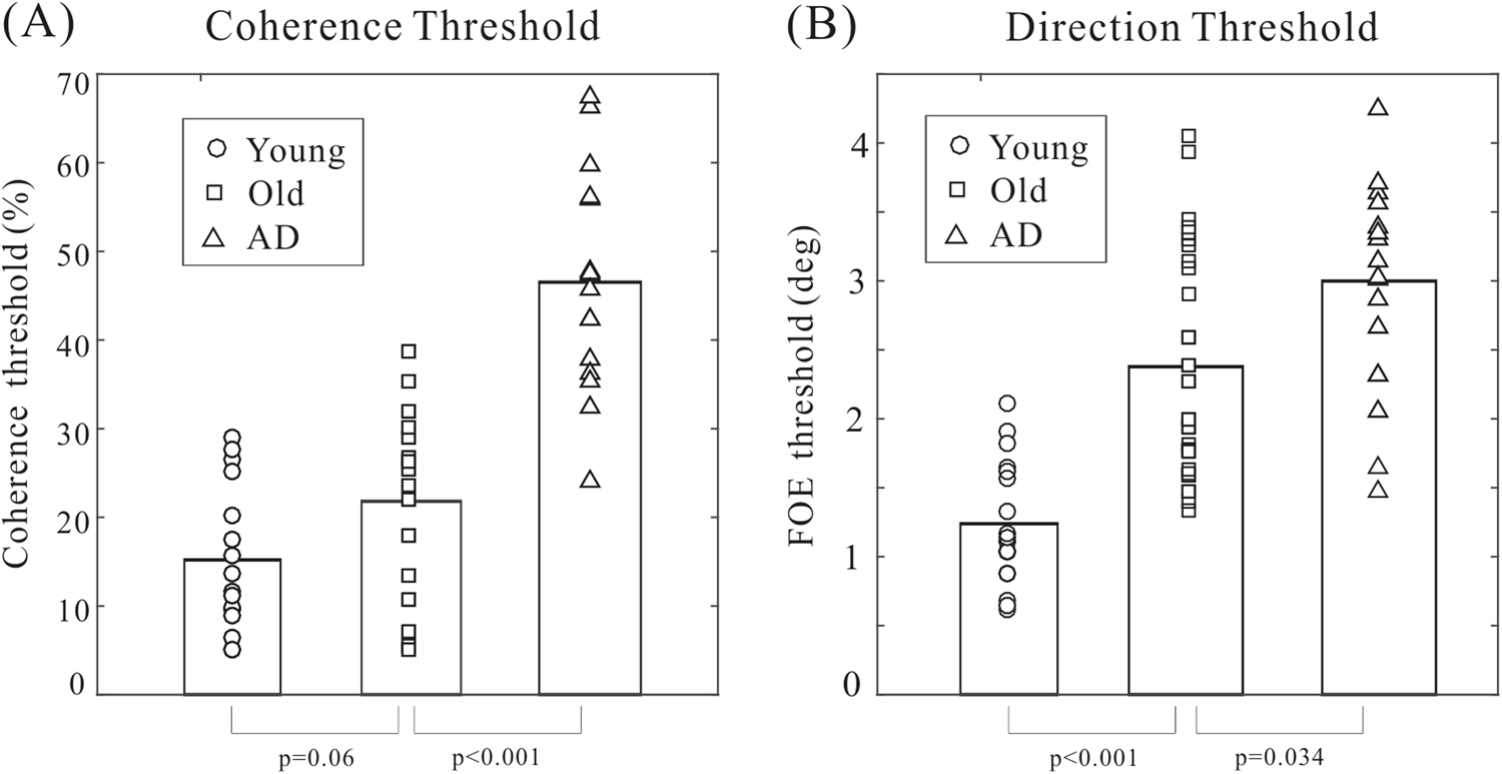
The coherence and direction thresholds measured for each participant. The scatter plot depicts individual data and the bar the mean thresholds averaged within each group.

We examined the coherence and direction thresholds for each subject in each group. First, we confirmed the previous findings of elevated coherence threshold in AD patients. As shown in Fig. 2A, there was a general trend of coherence threshold elevation from young participants to elderly individuals and to AD patients. One-way independent ANOVA analysis revealed that the coherence threshold differed significantly between groups (F(2,45) = 39.4, p < 0.001). We then performed a couple of post-hoc unpaired *t*-tests among groups. The mean coherence threshold was low in the young group (mean ± SEM: 15.2 ± 2.1%), and it increased modestly in the elderly group (21.8 ± 2.7%). The *t*-test analysis between these two groups suggested that the effects of normal aging was close to significance (*t*(30) = 1.95, p = 0.06). The coherence threshold further elevated during the transition to AD (46.5 ± 3.1%). The statistical analysis showed that there was a significant difference between AD patients and healthy elderly participants (*t*(30) = 6.1, p < 0.001).

Second, we found that the direction threshold exhibited a similar pattern of group differences (Fig. 2B). Specifically, the direction threshold increased gradually from the young group (1.24 ± 0.12 deg) to the elderly group (2.38 ± 0.21 deg) and to the AD group (3.0 ± 0.18 deg). We submitted the direction threshold to the one-way independent ANOVA analysis. The results indicated that there was a significant group effect (F(2,45) = 25.6, p < 0.001). We have also conducted several post-hoc unpaired *t*-test among groups on the direction threshold. The statistical analysis revealed that there were significant differences between the young and elderly groups (*t*(30) = 4.6, p < 0.001), and between the elderly and AD groups (*t*(30) = 2.2, p < 0.05). These results indicated that not only the coherence threshold but also the direction threshold were impaired in AD patients as comparing to healthy elderly controls.

**Figure 3:**
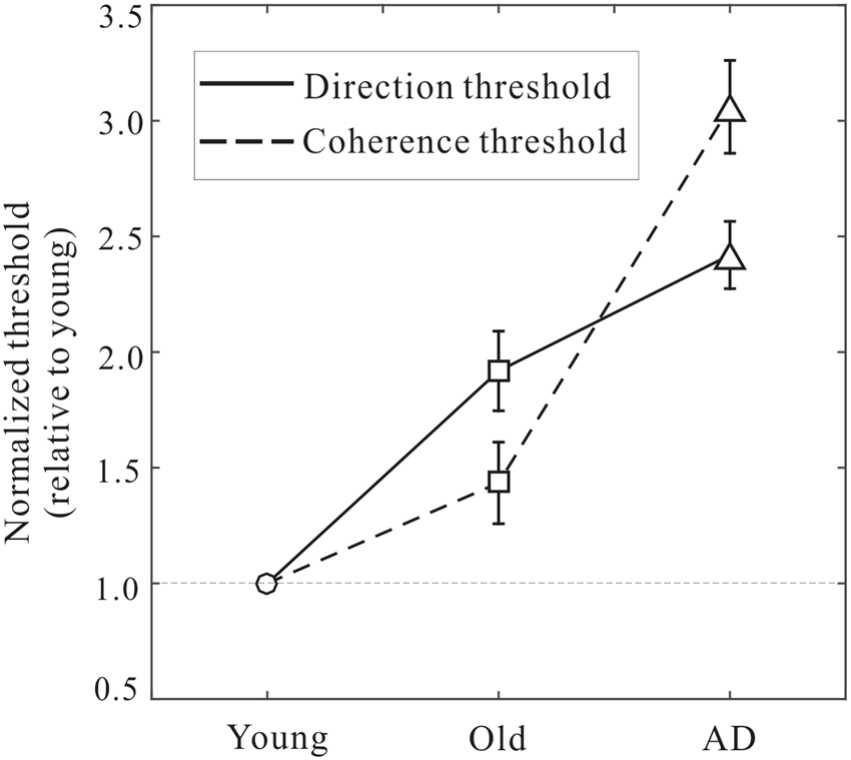
Mean normalized thresholds for the old and AD groups (relative to the mean of the young group). Results are shown for mean ± SEM averaged within each group for each type of threshold.

To explore the differences between the coherence and direction threshold impairments, we conducted the combined analysis in which we normalized each threshold value relative to its mean value in the young group. This allowed us to directly compare these thresholds across groups (AD vs. elderly) and tasks (direction vs. coherence). As depicted in Fig. 3, the combined analysis showed that, while both the direction and coherence thresholds exhibited elevations when transiting into AD, there were marked differences between these two types of impairments. More precisely, the increase of the coherence threshold from elderly to AD was more prominent than the direction threshold. We performed a two-way mixed model ANOVA analysis on the normalized thresholds (between-subject factor: group; within-subject factor: task). The analysis showed that there were significant effects on the group (F(1,30) = 38.7, p < 0.001) and on the task (F(1,30) = 239.7, p < 0.001), as well as on the interaction between the two factors (F(1,30) = 35.1, p < 0.001).

**Figure 4:**
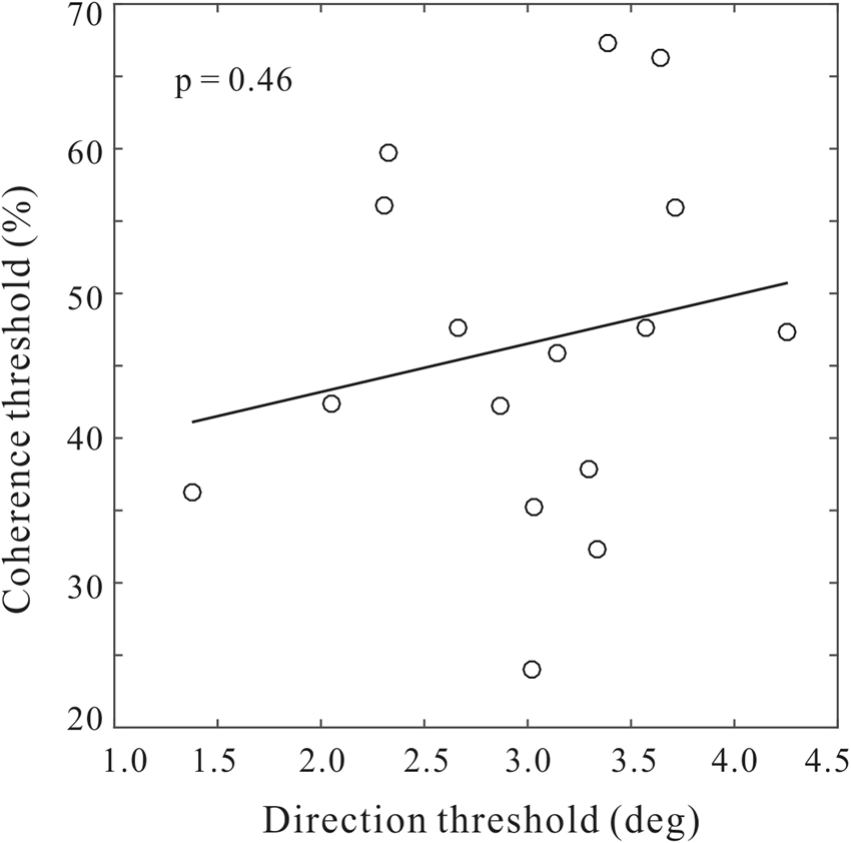
The Pearson correlation between the direction and coherence thresholds in AD patients.

We then attempted to determine whether and how the direction and coherence threshold impairments in AD were related to each other. To this end, we conducted the Pearson’s linear correlation analysis between the two thresholds in AD patients (Fig. 4). We found the correlation coefficient between them was small and statistically non-significant (r(14) = 0.19, p = 0.46). In addition, the direction and coherence thresholds were also uncorrelated in both the elderly group (r(14) = −0.16, p = 0.56) and the young group (r(14) = −0.24, p = 0.38). These results indicated that these two threshold measurements are differentially impaired in AD patients.

## Discussion

Our results have several important implications and merits. First, we confirmed the previous findings of increased coherence threshold in AD patients as compared to healthy elderly controls^8,17,18^. We then extended these results by showing that the direction and coherence thresholds are differentially elevated (Fig. 3). The coherence and direction thresholds are two common measures of motion sensitivity in motion research^25^. The direction threshold probes the fine heading discrimination capability while the coherence threshold concerns the global integration ability of the visual system that discounts visual noise in the motion stimuli^26^. To the best of our knowledge, no studies so far have made direct comparisons between the impairments of these two forms of thresholds in AD patients.

One novel aspect of the current study was that we have bridged this knowledge gap and explored whether these two forms of thresholds are uniformly or independently impacted by AD pathology. We reasoned that, if these two thresholds reflect shared cortical impairments, then they should be correlated in AD patients. Conversely, if these two thresholds reflect independent visual processing deficits, then they should not be correlated in AD patients. In our data, we did not find significant correlations between the two thresholds in AD patients (Fig. 4). This suggests that the second prediction is more likely to be true. The independence of the coherence and direction thresholds implicates that these two measures might reflect distinct visual cortical processing deficits^31–33^. This means, it will be of great advantage for future studies to combine these two measures to provide better perceptual biomarkers for early AD detection.

Another important aspect of the study is that we found the tendency of selective motion impairments in AD and aging (Fig. 3). While AD patients showed significant impairments in both the direction and coherence thresholds relative to the elderly controls, the impairments were more pronounced in the coherence threshold than the direction threshold. In contrast, aging was associated with greater impairments in the direction threshold than the coherence threshold, as compared with young participants. These results are consistent with the notion that AD patients appeared to have reduced global motion processing^34,35^ and the evidence that elderly subjects mainly suffer from deteriorated heading discrimination performance linked to spatial orientation deficits^36,37^. Since AD patients are often older adults, they are prone to aging effects irrespective of AD; it is thus desirable to have a perceptual biomarker that could dissociate AD from aging. In our data, AD and aging impaired the direction and coherence thresholds to a different extent, and this indicates that AD could be distinguished from aging by this differential pattern of motion deficits. Since the two measures of motion sensitivity reflect distinct cortical processing, our results of differential motion deficits in AD and aging suggest that AD may be associated with deficits in visual cortical processing which are not yet (or less) impaired in the brains of normal aging adults^38,39^. Identifying the behavioural and neural uniqueness of AD relative to normal aging is tremendously advantageous because it might lead to better identifications of the transition from aging to AD.

The current study had several limitations and considerations to be noted. First, for the sake of the constraints on the length of the experiments (especially for sensitive subjects like elderly and AD patients), we chose not to vary too many parameters (e.g., the heading speed, the dot density) in a single experiment. These motion parameters might influence the estimation of the coherence and direction thresholds per se. However, we consider that they are less likely to affect the results of threshold impairments. For example, a recent study by Li and Bremer^37^ showed that the decrements of heading performance with aging were robust across systematic variations of the presentation time and the number of dots in the display. Future studies will be needed to ensure that the current results are similarly robust to parameter variations in AD patients. Secondly, it should be noted that, while most previous studies on motion perception in AD focused mainly on the coherence threshold^5–8,17,18^, there was one study which examined the properties of the heading direction threshold in AD^6^. In that study, the goal was to compare the effects of the spatiotemporal compositions of the optic flow stimuli on the heading direction thresholds. The authors found that, relative to elderly controls, AD was associated with poorer heading direction thresholds only at lower temporal periodicity, i.e., the AD and age-matched elderly controls were not substantially different in the other temporal periodicity conditions. The heading stimuli of that study were a field of dots moving radially in a flat plane (without 3D depth information as we had in our study). As a consequence, the reported direction thresholds (e.g., in the young group) was about five times larger than the typical range of 1°–2° using the 3D cloud stimuli^21–24^. Due to these methodological differences, it is difficult to draw direct comparisons between their results and the current data.

## Data Availability

The data in this study is available upon reasonable request to the corresponding author.
